# Light Absorption in Nanowire Photonic Crystal Slabs and the Physics of Exceptional Points: The Shape Shifter Modes

**DOI:** 10.3390/s21165420

**Published:** 2021-08-11

**Authors:** Simeon Trendafilov, Jeffery W. Allen, Monica S. Allen, Sukrith U. Dev, Ziyuan Li, Lan Fu, Chennupati Jagadish

**Affiliations:** 1 Air Force Research Laboratory, Munitions Directorate, Eglin AFB, Valparaiso, FL 32542, USA; simeon.trendafilov.ctr@us.af.mil (S.T.); monica.allen.3@us.af.mil (M.S.A.); sukrith.dev.1@us.af.mil (S.U.D.); 2 Department of Electronic Materials Engineering, Research School of Physics and Engineering, The Australian National University, Canberra, ACT 2601, Australia; ziyuan.li@anu.edu.au (Z.L.); lan.fu@anu.edu.au (L.F.); chennupati.jagadish@anu.edu.au (C.J.)

**Keywords:** nanowire array, photonic crystal slab, exceptional point, infrared sensors

## Abstract

Semiconductor nanowire arrays have been demonstrated as promising candidates for nanoscale optoelectronics applications due to their high detectivity as well as tunable photoresponse and bandgap over a wide spectral range. In the infrared (IR), where these attributes are more difficult to obtain, nanowires will play a major role in developing practical devices for detection, imaging and energy harvesting. Due to their geometry and periodic nature, vertical nanowire and nanopillar devices naturally lend themselves to waveguide and photonic crystal mode engineering leading to multifunctional materials and devices. In this paper, we computationally develop theoretical basis to enable better understanding of the fundamental electromagnetics, modes and couplings that govern these structures. Tuning the photonic response of a nanowire array is contingent on manipulating electromagnetic power flow through the lossy nanowires, which requires an intimate knowledge of the photonic crystal modes responsible for the power flow. Prior published work on establishing the fundamental physical modes involved has been based either on the modes of individual nanowires or numerically computed modes of 2D photonic crystals. We show that a unified description of the array key electromagnetic modes and their behavior is obtainable by taking into account modal interactions that are governed by the physics of exceptional points. Such models that describe the underlying physics of the photoresponse of nanowire arrays will facilitate the design and optimization of ensembles with requisite performance. Since nanowire arrays represent photonic crystal slabs, the essence of our results is applicable to arbitrary lossy photonic crystals in any frequency range.

## 1. Introduction

Nanowires (NWs) and nanopillars have seen rapid evolution and many improvements in fabrication and materials over the past decade. Semiconductor nanowire devices have garnered much interest in the field of optoelectronics, with ternary III-V semiconductor NWs such as GaAs_x_Sb_1-x_ NWs finding particularly promising applications in photodetectors [1,2,3,4], photovoltaics/solar cells [5,6,7] and transistors [1,8]. These NWs have different accessible pathways for light absorption and carrier transport and exhibit prominent features, such as reduced reflectance, enhanced absorption efficiency, spectral selectivity and high quantum efficiency for carrier collection. Light absorption for individual NWs is well understood, and the responsible photonic modes have been identified as the well-known leaky waveguide modes of cylindrical rods [9,10,11]. Since light harvesting in single nanowire systems is limited, research has converged on much more capable nanowire arrays, which improve light collection and absorption through a collective response. Because of their geometry and periodic nature such structures naturally lend themselves to waveguide and photonic crystal mode engineering, leading to multifunctional materials and devices. Rapid development of techniques to synthesize vertical nanowire arrays and fabrication methods to transition them to practical devices have established them as a viable component for optoelectronic applications [12,13,14,15,16]. While fabricated devices have achieved competitive figures of merit [17], it is evident that optimization of nanowire array parameters to increase absorption of incident light is crucial for improved efficiency [18]. Here we show a path to take advantage of their unique properties by developing the theoretical foundation to design, engineer and optimize multifunctionality in nanowire array detector devices enabling enhanced use in multicolor imaging, single-photon detection, LIDAR, room-temperature IR detection, etc.

Nanowire array slabs exhibit resonant absorption peaks and significant effort has been dedicated to develop the theoretical foundation to tune their resonant properties and facilitate system design. While full wave 3D simulations give good agreement with experimental observations, they do not provide the physical underpinnings of the modes responsible for the resonances and lead to a somewhat scattergun approach in designing arrays with a desired photonic response. Models that define the underlying physics of the modes will lead to better understanding for design, optimization and prediction of performance of nanowire arrays. A standard practice has been to interpret the results from 3D simulations of photonic crystal slabs by relating their spectral features to the leaky modes of a single infinitely extended cylindrical rod [19,20]. As shown in [11], a close relationship exists between the leaky modes and Mie resonances of an infinite circular cylinder, and thus the Mie extinction/scattering/absorption point of view can also be used in conjunction with the eigenmodes. However, conceptually, the relevance of leaky waveguide modes of single nanowires within an array is not obvious. A periodic array of nanowires/rods is a 2D photonic crystal and thus any mode propagating within it is a bona fide Bloch photonic crystal mode. To provide a connection between the two types of modes, Fountaine et al. [20] advanced the view that for sufficiently large nanowire spacings, the photonic modes of an isolated nanowire are only slightly perturbed by the array “lattice”, analogous to the situation in atomic crystals. A mathematical treatment following similar ideas was used by Strumberg et al. [21] to derive a variation of the cylindrical rod waveguide eigenvalue equation that accounts for the nearest neighbor effects from the lattice array. While this equation correctly reduces to the eigenvalue equation for an individual nanowire in the limit where the lattice array period becomes infinitely large, this approach is only appropriate for dilute arrays where the interwire spacing is sufficiently large that most interaction consequences can be neglected. Furthermore, it does not fully and explicitly take into account the periodicity of the array. An alternative to using single nanowire waveguide modes is to use numerically calculated photonic crystal modes of a 2D lattice of infinitely extended cylinders. Any such models also need to take into account the coupling of modes to plane waves at the photonic crystal slab interface through overlap integrals [22,23,24,25]. For the finite longitudinal wave vectors involved, numerical treatment of the modes is used, since analytical results are typically restricted to transverse plane propagation where additional symmetry arguments can be applied. Modal dispersion curves are obtained, and electric and magnetic field mode profiles at selected points are used to characterize the modes. It was established that a small number of photonic crystal modes are dominant contributors to the absorption within a given spectral range, leading Strumberg et al. [24] to name them key modes. However, despite numerous studies that have shed light on pertinent features of these key modes, a coherent physical perspective that gives a systematic view of their origin and attributes has remained elusive. Combinations of different approaches to mode description are often encountered complicating the analysis, which exemplifies the need for a single complete model that can describe and predict the behavior of nanowire arrays.

Note that the eigenmodes encountered in the nanowire structures under consideration are lossy modes (conducive to the goal of absorbing radiation) in a photonic crystal environment, where the density of states is high and mode interactions are abundant. The description of such non-Hermitian interactions is fairly well-developed and has given rise to the physics of exceptional points—branch point singularities in the parameter space of non-Hermitian systems at which eigenvalues and their corresponding eigenvectors coalesce. The crossing and anticrossing of energies (frequencies) and widths drew attention in the context of interacting unbound states in two level systems with numerous examples in nuclear and particle physics, optics and atomic physics [26]. It was eventually generalized and formalized as the physics of exceptional points [27,28,29]. Recently, there has been growing interest in exceptional points in the fields of optics and photonics [30], but so far in the theoretical description of resonant absorption of radiation in NW arrays, the phenomenon of exceptional points in the interactions of the photonic crystal lossy modes has not received proper attention. The treatment of the key modes from the point of view of exceptional points is the focus of this paper.

Exceptional points can be encountered in handling non-Hermitian matrixes such as the matrix appearing in the differential equation describing two coupled modes propagating along the axis of a 2D photonic crystal:

(1) $$\frac{d}{dz}\left(\begin{array}{c} a_{1}\\ a_{2} \end{array}\right)=-i\left(\begin{array}{cc} \beta_{1}-i\gamma_{1} & \delta \\ \delta & \beta_{2}-i\gamma_{2} \end{array}\right)\left(\begin{array}{c} a_{1}\\ a_{2} \end{array}\right),$$

where $a_{1,2}$ are the modal amplitudes, $\beta_{1,2}$ are the propagation constants of the decoupled modes, $\gamma_{1,2}$ are their decay rates and δ is the coupling coefficient. The complex eigenvalues of the matrix are:

(2) $$\lambda_{\pm }=\beta_{a}-i\gamma_{a}\pm \sqrt{\delta^{2}+{\left(\beta_{∆}+i\gamma_{∆}\right)}^{2}},$$

where $\beta_{a}=\left(\beta_{1}+\beta_{2}\right)/2$, $\gamma_{a}=\left(\gamma_{1}+\gamma_{2}\right)/2$, $\beta_{∆}=\left(\beta_{1}-\beta_{2}\right)/2$ and $\gamma_{∆}=\left(\gamma_{1}-\gamma_{2}\right)/2$. Here the matrix elements are functions of system parameters such as period, NW diameter, refractive index and frequency. An exceptional point occurs when the expression under the square root in Equation (2) becomes equal to zero. Unlike the accidental degeneracies of Hermitian operators (diabolical points) where independent eigenvectors are present with a common eigenvalue, at an exceptional point the eigenvectors also coalesce along with the eigenvalues. The proximity and location of exceptional points are closely connected to the phenomenon of level repulsion. In the complex plane of a system parameter, the square root in Equation (2) will generally approach zero at a complex parameter value (unless an extreme coincidence or special tuning of the other system parameters occurs), and when plotting the eigenvalues as a function of the real part of the parameter, either the real or imaginary parts of the two eigenvalues will avoid each other. This will manifest itself as either repulsion of levels or repulsion of widths depending on the location of the exceptional point in the complex parameter plane. If the exceptional point lies below the real axis, level repulsion (width crossing) occurs, and if the exceptional point lies above the real axis, width repulsion (level crossing) occurs [27]. Eigenvalue repulsion entails strong coupling and hybridization between states, making it of great interest in photonics. As we show below, such hybridization is at the center of the absorption resonances in NW photonic crystals.

## 2. Materials and Methods

We are interested in the origin of the resonance properties in NW array slab absorption and the fundamental details of the underlying physics. Some simplifying assumptions are made to elucidate basic rules of the system’s physical behavior. The goal is to relate absorption peaks from 3D-driven simulations of a photonic crystal slab suspended in air to modes of an infinitely extended 2D photonic crystal. The photonic crystal lattice is a square array with pitch, P, composed of cylindrical rods with diameter, D. Normal incidence is considered, and as a consequence the dipolar modes that couple to incoming plane waves are doubly degenerate. We remove effects arising from material dispersion and set the complex refractive index of the nanowires to one of three constant values. The essential difference between these values is the amount of losses they introduce into the system. These simulations are associated with tuning the absorption peaks of GaAs_0.94_Sb_0.06_ NW arrays, and the constant values are selected so that the maxima of the primary absorption peaks for 100, 120 and 150 nm diameter NW arrays with 400 nm pitch maintain their spectral positions at 560, 615 and 720 nm, respectively. The refractive indexes chosen, $n_{1}$ = 4.05 + 0.370i, $n_{2}$ = 3.97 + 0.290i and $n_{3}$ = 3.81 + 0.143i, are the refractive indexes of the ternary compound GaAs_0.94_Sb_0.06_ at the given wavelengths and are obtained by linear interpolation from the binary parameters. The refractive index values for the binary compounds GaAs and GaSb are extracted from measured data [31,32].

Simulation was carried out using the commercial software package Comsol Multiphysics^®^. The simulation domain is a unit cell with continuity periodic boundary conditions in the transverse dimensions. In the 3D simulations, the domain is terminated with perfectly matched layers (PMLs) in the longitudinal dimension and the source radiation is a plane wave at normal incidence. The slab thickness (length of the NWs) is 2 μm. In the 2D simulations, a complex propagation constant, β, is computed as the eigenmode eigenvalue at a given input wavelength/frequency. Dispersion plots are presented as effective refractive index $n_{eff}=\beta /k_{0}$ as a function of wavelength, $\lambda_{0}$, where $k_{0}=2\pi /\lambda_{0}$.

## 3. Results and Discussion

**Figure 1:**
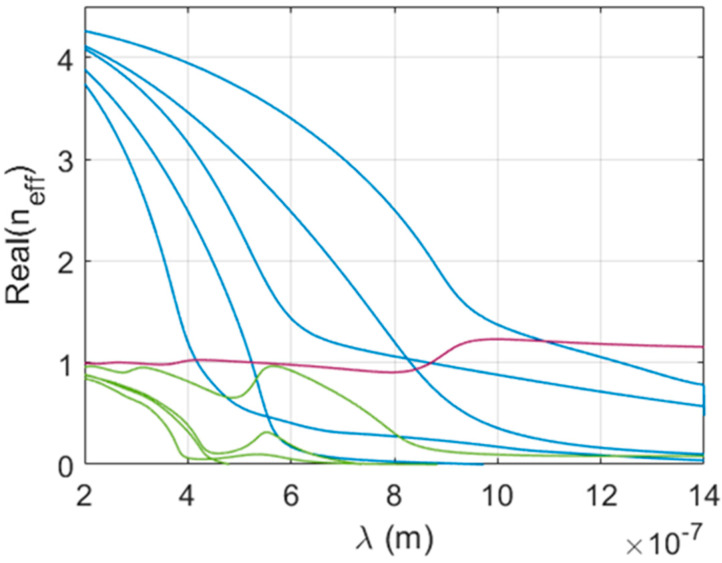
Dispersion diagram for a square array of circular rods in air. Array parameters: P = 400 nm, D = 150 nm and $n_{rod}=4.37+0.58i$. Fiber modes colored blue and photonic crystal modes colored green, except for ${\mathrm{PC}}_{0}$ colored purple.

We can generally divide the modes of a 2D photonic crystal consisting of high refractive index rods into two types based on the primary location of power flow as follows: (i) modes where most of the power flows along the rods/nanowires are referred to as fiber modes, and (ii) modes for which most of the power flows through the embedding medium (air) surrounding the rods are referred to as photonic crystal modes. We emphasize that both types of modes are proper photonic crystal modes regardless of the terminology used. A typical dispersion diagram for a square array of highly lossy nanorods is shown in Figure 1. At higher frequency (shorter wavelength), the effective refractive index of the fiber modes converges to that of the nanorod material ($n_{eff}\to 4.37$) and the modal properties converge to those of the corresponding single fiber modes, from which we derive their names. Fiber modes have a cutoff at lower frequency (longer wavelength). The effective refractive index of the photonic crystal modes in the high frequency (short wavelength) limit tends to that of the embedding material, i.e., air. These modes also have a cutoff at lower frequency (longer wavelength) with the exception of the fundamental mode at the gamma point (i.e., in the case of zero transverse wave vector). If array parameters are such that we can effectively homogenize the physics at longer wavelengths, their cutoffs are near the different bands in the empty lattice approximation [33]. The fundamental photonic crystal mode ${\mathrm{PC}}_{0}$ deserves special attention. In the high frequency limit, the effective refractive index of ${\mathrm{PC}}_{0}$ fluctuates around that of the embedding material; in the low frequency limit, the effective refractive index of ${\mathrm{PC}}_{0}$ approaches that of the average medium in the empty lattice approximation. It is connected to the lowest TM band and has no cutoff at the gamma point, which is the relevant phase shift at normal incidence. This ${\mathrm{PC}}_{0}$ mode, by interacting and mixing with particular fiber modes, leading to either avoided crossings or avoided line widths, is responsible for all major absorption peaks. Its distinguishing feature is its excellent coupling to external plane waves. The mode ${\mathrm{PC}}_{0}$ has the necessary dipolar symmetry [18,19], and its transverse electric field is fairly uniform within the unit cell. Hence, its overlap integral with a plane wave is high. The effective refractive index of ${\mathrm{PC}}_{0}$ is always close to unity and it produces very little reflection due to impedance mismatch (Fresnel reflection). Most of the coupling from incoming radiation takes place in this particular mode except when rod diameters are on the order of the pitch, which results in very high fill rates. However, a high fraction of the ${\mathrm{PC}}_{0}$ mode energy flow is in air and therefore this mode is not very lossy. In contrast, the fiber modes have very high losses, but for practical rod diameters these modes couple poorly to plane waves, even when the coupling is not precluded by mode symmetry. Nevertheless, when the dispersion relation of fiber modes brings them close to the fundamental mode ${\mathrm{PC}}_{0}$ in a given spectral range, symmetry permitting, they interact with the fundamental mode and the resulting hybridized modes have both good coupling to plane waves and high losses. This interaction is not particularly obvious in customary dispersion diagrams that plot only the real part of the eigenvalue for the mode, when the mode curves cross in the case of avoided line widths. The crucial fiber modes that produce the desired interactions, as expected, are the ${\mathrm{HE}}_{1m}$ fiber modes.

**Figure 2:**
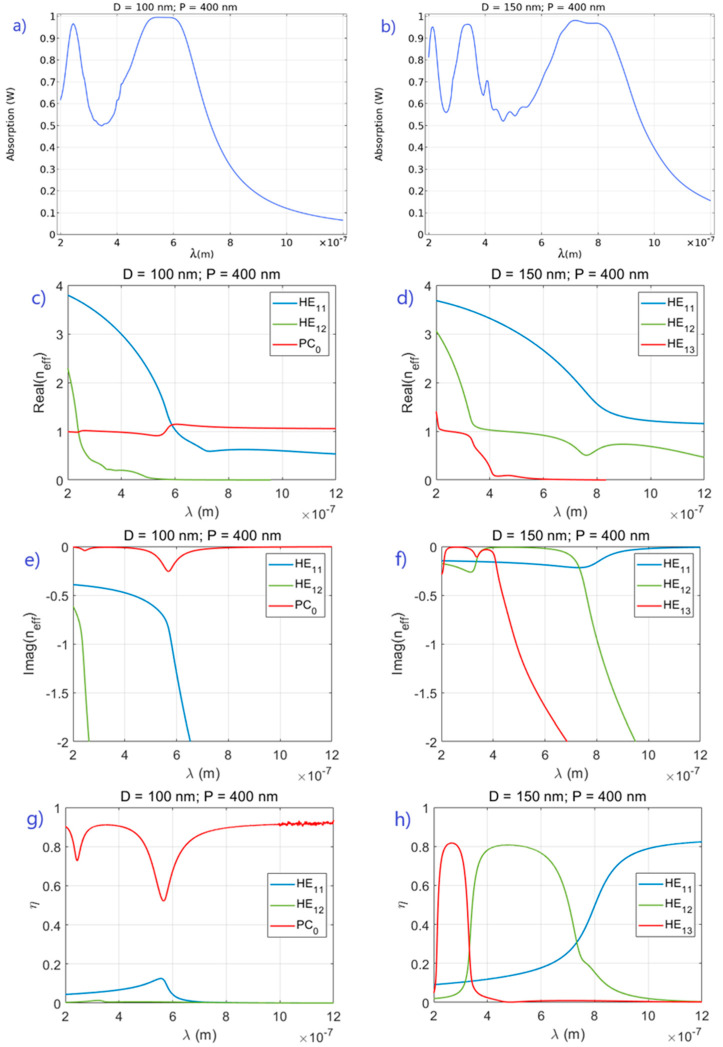
An example of avoided width crossings for NW refractive index $n_{1}$ = 4.05 + 0.370i (**a**,**c**,**e**,**g**); avoided frequency crossings for NW refractive index $n_{3}$ = 3.81 + 0.143*i* (**b**,**d**,**f**,**h**).

In Figure 2 we illustrate the two fundamental types of mode interaction: (a), (c), (e) and (g) show avoided line widths (exceptional points above the real frequency axes) for 100 nm NWs with refractive index $n_{1}$, and (b), (d), (f) and (h) show avoided levels (exceptional points below the real frequency axes) for 150 nm NWs with refractive index $n_{3}$. Figure 2a,b shows the absorption spectra from 3D simulations of the two photonic crystal slabs. In NWs with D = 100 nm, the photonic crystal modes responsible for the observed absorption peaks are the ${\mathrm{HE}}_{11}$ and ${\mathrm{HE}}_{12}$ modes through their interaction with the ${\mathrm{PC}}_{0}$ mode. The real and imaginary parts of the modal effective refractive indices from 2D eigenvalue simulations are plotted in Figure 2c,e and show the crossing of levels and anticrossing of widths. The centers of the absorption peaks coincide with the local maxima in the absolute value of $Imag\left(n_{eff}\right)$ for the ${\mathrm{PC}}_{0}$ mode. These maxima also coincide with the minima in the overlap integral for the ${\mathrm{PC}}_{0}$ mode shown in Figure 2g, so they seem to be at the frequencies of strongest interaction between the modes. In the NWs with D = 150 nm, the dispersion diagram patterns, presented in Figure 2d,f, are quite different. For all three fiber modes, ${\mathrm{HE}}_{11}$, ${\mathrm{HE}}_{12}$ and ${\mathrm{HE}}_{13}$ that underpin the three absorption peaks in the spectral interval considered, levels repel by the ${\mathrm{PC}}_{0}$ mode, so that the fundamental mode breaks up. In this case, it is the imaginary parts of the effective refractive indices that cross as well as the overlap integrals representing the coupling efficiency, as shown in Figure 2h. Overlap integrals are defined as:

(3) $$\eta =\frac{\|\langle E_{inc}{|E}_{m}\rangle \|{}^{2}}{\left\|\langle E_{inc}{|E}_{inc}\rangle \|\|\langle E_{m}{|E}_{m}\rangle \right\|},$$

where $E_{inc}$ and $E_{m}$ are the incident (linearly polarized plane wave) and the photonic crystal mode fields, and $\langle A|B\rangle =\int da\;A\;\cdot \;B^{*}$ with integration taken over the 2D unit cell [22].

**Figure 3:**
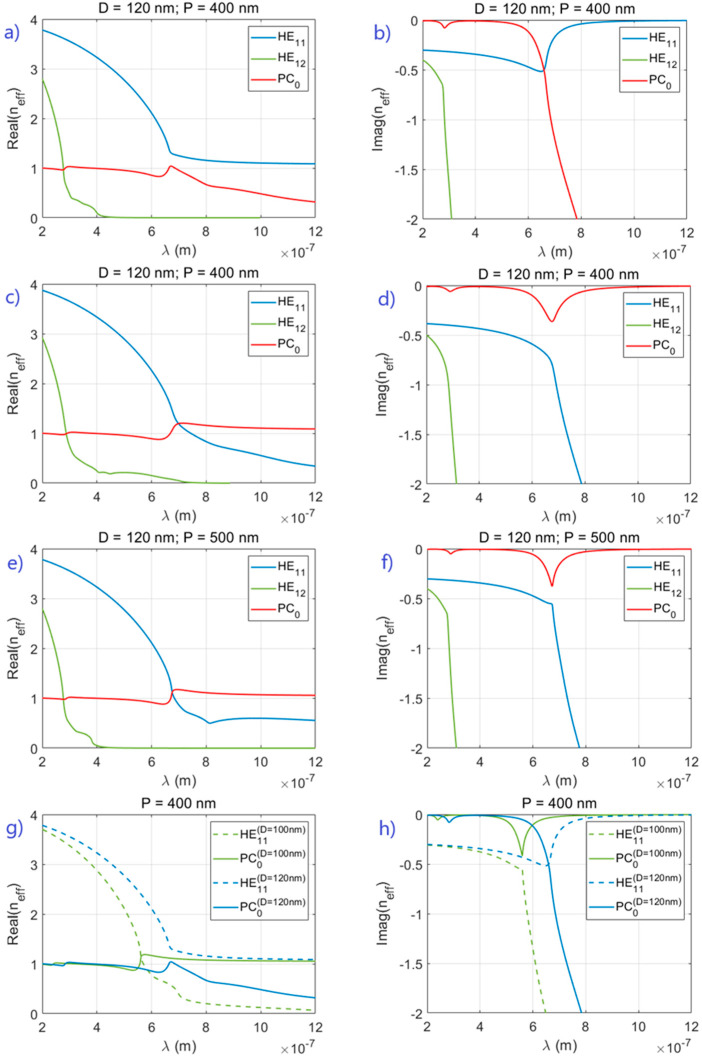
Switching between repulsion of levels and repulsion of widths by changing material and geometry parameters: (**a**,**b**,**e**–**h**)—refractive index $n_{2}$; (**c**,**d**)—refractive index $n_{1}$. Geometry parameters as given on plots.

Next, we manipulate the position of an exceptional point by changing basic NW array parameters, viz., NW refractive index, array periodicity/pitch and NW diameter. Our starting point is an array of NWs with refractive index $n_{2},$ diameter D = 120 nm and pitch P = 400 nm. These parameters lead to the coupling of the ${\mathrm{HE}}_{11}$ fiber mode with the fundamental mode ${\mathrm{PC}}_{0}$ resulting in a case of avoided levels, as shown in Figure 3a,b. Shifting the exceptional points of the mode crossings of higher order ${\mathrm{HE}}_{1m}$ modes with ${\mathrm{PC}}_{0}$ requires larger variation of parameters, so we focus on ${\mathrm{HE}}_{11}$. We begin by analyzing the effect of NW refractive index. A change in the NW refractive index from $n_{2}$ to $n_{1}$ moves the exceptional point above the real frequency axis, and the interaction regime switches to level crossing (avoided line widths), as seen in the contrast between Figure 3a,b and Figure 3c,d.

This refractive index change from $n_{2}$ to $n_{1}$ leads to higher losses, and the observed change in regime is consistent with the results in [27]. Then we examine the effect of changing the periodicity or pitch, which affects the coupling between the modes. Increasing the pitch from 400 to 500 nm (NW refractive index kept equal to $n_{2}$) moves the exceptional point above the real frequency axis, shown in Figure 3e,f, an effect similar to increasing the losses. Finally, we explore the effect of NW diameter on shifting the location of the exceptional point in the complex frequency plane. The results from varying the NW diameter are presented in Figure 3g,h, (NW refractive index equal to $n_{2}$). In addition to changing the regime from avoided levels to level crossing, changing the NW diameter has the additional effect of strongly shifting the frequency of the absorption resonances. Thus, we demonstrate that when we implement the standard methods of tuning an exceptional point, namely changing the mode losses and the interaction strength between modes by varying geometrical and material properties of the NW arrays, the interacting 2D photonic crystal modes behave as expected around an exceptional point. Overall, for this particular nanowire array system, exceptional points for this system in the complex frequency plane will become real between $n_{1}$ and $n_{2}$ (at D = 120 nm and P = 400 nm), between P = 400 nm and P = 500 nm (at $n_{2}$ and D = 120 nm) and between D = 100 nm and D = 120 nm (at $n_{2}$ and P = 400 nm).

## 4. Conclusions

We have shown for the first time that the modal interactions in photonic crystal slabs of lossy rods, which underpin the photonic response of semiconductor NW array optoelectronic devices, are a manifestation of the physics of exceptional points. Due to improved understanding and control of nanowire fabrication process parameters [1,2,3], such as growth temperature and material composition, the locations of the exceptional points in the complex frequency plane can be easily manipulated and experimentally achieved in the future by changing material and geometrical parameters such as NW refractive index, array pitch and NW diameter. Consequently, this would enable and enhance the use of nanowire arrays in molecular sensing, spectroscopy and light detection applications in which operation at the exceptional point results in enhanced sensitivity to small perturbations [34,35,36]. This demonstrates how exceptional point analysis can be used to optimize performance using NW array parameters and tune the photonic response. The exceptional point could be positioned on or very close to the real frequency axes in order to access the benefits of coalescing modes. A photonic crystal slab has a plethora of interacting modes with widely varying properties, which allows engineering of a broad variety of responses. We examined the interaction between a very lossy doubly degenerate fiber mode and the very leaky doubly degenerate fundamental mode of the photonic crystal at normal incidence. At oblique incidence, these degeneracies break up, but as expected and verified by simulations, the physics essentially remains the same. The anomalous parameter dependence in the response of the inherently lossy photonic crystal slab system around exceptional points opens the door to novel ways of tailoring frequency, polarization and phase response. The results from the physics of exceptional points can be applied to 2D photonic crystal modes to explain the observed widely varying dispersion bands that underpin resonant absorption in NW array slabs. Further, this new insight will also open up possibilities for exciting new research directions to tune different responses, enabling pervasive technologies such as multispectral and multifunctional apertures with super pixel arrays for detection and imaging.

## Data Availability

Not applicable.
